# Evaluating a Response to a Canine Leptospirosis Outbreak in Dogs Using an Owner Survey

**DOI:** 10.3390/vetsci12020119

**Published:** 2025-02-02

**Authors:** Sierra Villanueva, Cord Brundage

**Affiliations:** 1 Animal and Veterinary Science Department, California State Polytechnic University, Pomona, CA 99701, USA; 2 Biology Department, University of Wisconsin, La Crosse, WI 55601, USA

**Keywords:** canine leptospirosis, owner survey, outbreak, elective vaccinations

## Abstract

Leptospirosis is a bacterial zoonotic disease that spreads through contaminated soil and water or directly from infected animals through urine. Although animal-to-human transmission is low, humans are most susceptible to contracting leptospirosis from these contaminated sources. This makes leptospirosis a public health concern, and therefore it is important to control these bacteria from spreading into the environment. A survey targeting Los Angeles County communities, in which a 2021 leptospirosis outbreak occurred, was sent out via groups on the online platforms Instagram and Facebook to gather dog and owner demographics. With 92 (90.2%) respondents having a primary veterinarian, it could not be determined what caused certain owners to have a greater vaccination rate than those who did not (n = 10; 9.8%). Overall, 69 respondents (68%), regardless of whether they had a primary veterinarian or not, reported not knowing of canine leptospirosis and 79 (77%) not knowing the signs to look for or that it is zoonotic. These data help provide a basis in terms of the status of dog owners’ knowledge of leptospirosis and how to begin to inform dog owners better about preventatives for this disease.

## Simple Summary

Leptospirosis is a bacterial zoonotic disease that spreads through contaminated soil and water or directly from infected animals through urine. In January 2022, a survey targeting Los Angeles County communities, in which a 2021 leptospirosis outbreak occurred, was sent out via groups on the online platforms Instagram and Facebook. A total of 102 dog owners completed the survey, and 90% reported having a primary veterinarian, 68% of the respondents were unaware of the outbreak, and 77% were unfamiliar with the signs and risks associated with leptospirosis. These data provide some insight into dog owners’ awareness and perceptions of leptospirosis and the 2021 outbreak.

## 1. Introduction

Leptospirosis is a zoonotic bloodborne infectious disease caused by *Leptospira* spp. bacteria [1]. These flagellated, spiral-shaped, spirochete, Gram-negative bacteria can infect humans and most mammals, resulting in organ failure and death [2]. *Leptospira* spp. shed from the urine and/or tissues from host species and can persist in water or soil and remain pathogenic for over 150 days [3]. These bacteria enter through the eyes, mucus membranes, and skin wounds and target the kidneys, lungs, liver, and/or meninges of infected individuals [2,4]. Pathogenic *Leptospira* spp. are able to evade host immune complement defenses and secrete phospholipases or sphingomyelinases that degrade host cell membranes [4]. The clinical signs of leptospirosis onset generally develop within a few days of infection, although they can extend for up to 30 days [2]. The shedding of *Leptospira* spp. from the urine of infected humans and animals has a short latency and is not always associated with clinical signs [5].

Dogs are commonly affected, especially if they have access to water or soil where infected wildlife may have shed these bacteria. The signs of leptospirosis can vary in degree in dogs. Factors that influence its severity include the infecting strain, geographical location, and the immune response of the host. Experiments have shown that altering the periplasmic flagella reduces the virulence of Leptospira, which indicates that motility is an important factor in determining its pathogenicity [6]. The case-fatality rate for leptospirosis in dogs can be as high as 20%. Younger dogs or dogs with a less developed or weakened immune system are at greater risk of serious effects [6]. A review of veterinary medical database case data from the 1970s indicated that the highest prevalence of leptospirosis in dogs (296.5 cases per 100,000 dogs) occurred in 1971 prior to the onset of the leptospirosis vaccination [7,8]. The decline in its prevalence in the 1980s (<10 cases per 100,000 dogs) is accredited to public awareness and vaccine efforts [8]; however, the prevalence of leptospirosis in dogs has continued to increase since the 1980s, with >150 cases per 100,000 dogs from 2004 to 2009 [7,8]. Clusters of these increasing numbers of positive cases have been reported around Texas, California, and the upper Midwest, indicating certain areas in the United States are also disproportionately affected [9]. 

Vaccines for leptospirosis are elective and recommended depending on the exposure risk to a dog [10]. Where dog owners acquire health information is a factor in leptospirosis vaccination rates. Sometimes, owners do not know the questions they need to ask their veterinarians, which has been shown to heavily influence owner decision-making [11]. If owners are not aware of certain conditions, outbreaks, or medications (including vaccines), then they are dependent on information from other sources, either credible or not. Possible sources of information other than their primary veterinarian would include animal shelters, websites, and community health officials. The sources of information that dog owners use should keep updated information. By having consistent updated information, dog owners can make educated choices regarding their dogs’ health. 

An outbreak of leptospirosis began in July 2021 in Los Angeles County and the surrounding communities [12]. From this outbreak, there were 201 confirmed cases that resulted in 13 deaths. Of the cases with data available, a 55% (91/164) majority of the infected dogs were hospitalized. Data on possible exposure sites were available for 154 cases and showed that 66% of the dogs possibly contracted the disease from social gatherings at places like daycares or boarding facilities and 49% (51/105) contracted it at dog parks. Owners also mentioned hiking, visiting beaches, and walking around the neighborhood [12]. Los Angeles County officials reported that the suspected serovar for this specific outbreak was Canicola [12]. This serovar is known to have dogs as its primary reservoir, which made this outbreak heavily reliant on dog-to-dog transmission [13]. According to Los Angeles County health officials, as of March 2022, the outbreak has concluded. However, dog owners in the county are encouraged to vaccinate if they attend places where dogs gather, such as parks, training facilities, groomers, and boarding facilities [12]. 

In response to this outbreak, we published a survey asking Los Angeles County dog owners to answer a series of background questions to gather demographics on both owners and dogs in that community after the outbreak. The goal was to understand the knowledge of and familiarity with canine leptospirosis the Los Angeles County community had to improve responses in the form of vaccinations to potential outbreaks in the future. We also wanted to see whether owners with at-risk dogs and access to credible information from a primary veterinarian reported higher leptospirosis vaccination rates than other owners.

Since it has been shown that there are a multitude of factors that go into acquiring and taking care of a dog, it was important to also consider the owner demographics. Some owners put thought, research, and experience behind their decision, while others make it on impulse. Often, this can also influence the care of the dog, like gauging the health risks of certain diseases like leptospirosis [14]. The number or kind of household members, type of accessible housing, education level, prior dog ownership, age of both the dog and the owner, and behavior of the dog have all been demonstrated to be factors that work together in deciding dog ownership [14]. Specifically, owners with lower education and who are younger in age tended to have increased odds of dog ownership. Most owners were also found to spend little time (less than a week) researching prior to adopting their dogs. The most popular sources found when research was carried out were internet searches, books, breeders, and friends and family [14]. 

## 2. Materials and Methods

The Cal Poly Pomona Institutional Review Board reviewed and approved this research involving human subjects under protocol IRB 21–200. 

### 2.1. The Survey

A survey presented to dog owners in Los Angeles County communities was based on 47 total questions divided into 5 sets (refer to Supplementary Materials). All questions were marked as mandatory to avoid skipped questions unless otherwise specified in the survey. Examples of questions that could be skipped included the age of children in the home (if there were none), why the respondent chose to vaccinate their dog (if the dog was unvaccinated), and why the respondent chose to not vaccinate their dog (if their dog was vaccinated). The first question reviewed the purpose and conditions of the survey and gained participants’ consent to participate in the survey. The first subsequent set of questions obtained owner demographics such as gender, age range, whether they were the primary owner, and whether they were a resident of a canine leptospirosis outbreak area (Los Angeles County). Certain questions were clarified in the answer choices if necessary. For example, advanced dog owners were those who had had multiple dogs over numerous years, intermediate dog owners were those who had had a couple of dogs over a few years, and beginner dog owners were those who had a dog for the first time. 

The second set of questions identified basic information about one (of the) dog(s) owned (if multiple dogs were owned). These questions asked about the sex, age range, social interactions, and primary health provider for the dog. Animal husbandry was the third question set. These questions asked about the dog’s access outside of the home, how the dog was housed, and whether the dog partook in common social activities, like grooming or boarding. The fourth question set established the health status of the dog. These questions covered whether the dog was currently on core vaccinations, had any emergency visits, had annual wellness exams, or suffered from chronic conditions. The background information for the dogs was taken partially from the survey research conducted on hearing impairments and demographics in canines [15]. The final question set identified the owners’ knowledge of leptospirosis. The respondents were asked whether they were familiar with the disease, whether their dog was vaccinated against leptospirosis and why, whether they knew the signs of leptospirosis, and what their source of information was. 

The entire survey was accessed on SurveyMonkey (SurveyMonkey Inc., San Mateo, CA, USA). Once the survey was created, the link was made live on the Facebook page “Los Angeles Pet Services” with approval from the page administrator. The survey posting stipulations allowed for self-promoting posts. The link was also made live on Instagram. Links to the survey were added to the account page in the biography section and to the promotional “story” feature. The timeframe for taking the survey was 14 days from 1 January 2022 to 15 January 2022. The link was closed, and the results were analyzed after this time. Any results that did not contain a fully completed survey were deemed incomplete and discarded. 

### 2.2. Statistical Analysis

The results were sorted into four main groups based on the primary veterinarian question: (a) primary veterinarian and contact with outside animals, (b) primary veterinarian and no contact with outside animals, (c) no primary veterinarian and contact with outside animals, and (d) no primary veterinarian and no contact with outside animals. These groups were made using the filter system available in SurveyMonkey. The data collected were analyzed based on the number of respondents for each grouping that was adequately represented. A chi square analysis was performed using SPSS on two of the groups: (a) primary veterinarian and contact with outside animals and (b) primary veterinarian and no contact with outside animals. 

## 3. Results

### 3.1. Owner Gender and Age

Over 300 voluntary respondents started the survey; however, only 102 fully completed the survey and were used in this analysis. Completion of the survey required the participants to reside in Los Angeles County and own at least one dog. We let the respondent define/determine for themselves whether they were a dog owner. Surveys for which there were partial results at any point were considered to represent the respondent retracting their consent, and therefore these responses were excluded from the analysis. Of the respondents, 89% (91/102) were women, 10% (10/102) were men, and 1% (1/102) were non-binary. The ages of the respondents were 13% (13/102) 18–24 years, 43% (44/102) 25–34 years, 12% (12/102) 35–44 years, 16% (16/102) 45–54 years, 12% (12/102) 55–64 years, and 5% (5/102) 65 years or older (see Table 1). 

### 3.2. Dog Sex and Age

Participants with more than one dog were asked to self-select only one dog from their household to complete the survey without considering the dog’s breed or any other factors. Consequently, the number of animals addressed in the survey aligns with the number of participants. Of the 102 dogs, 52% (53/102) were altered males, 35% (36/102) were altered females, 9% (9/102) were unaltered males, and 4% (4/102) were unaltered females. Furthermore, the ages of the dogs were as follows: 37% (38/102) were 0–2 years, 11% (11/102) were 3–4 years, 13% (13/102) were 5–6 years, 10% (10/102) were 7–8 years, 13% (13/102) were 9–10 years, and finally, 17% (17/102) were 11 years or older (see Table 2). 

### 3.3. Dog Ownership Level and Prior Dog Research

**Figure 1:**
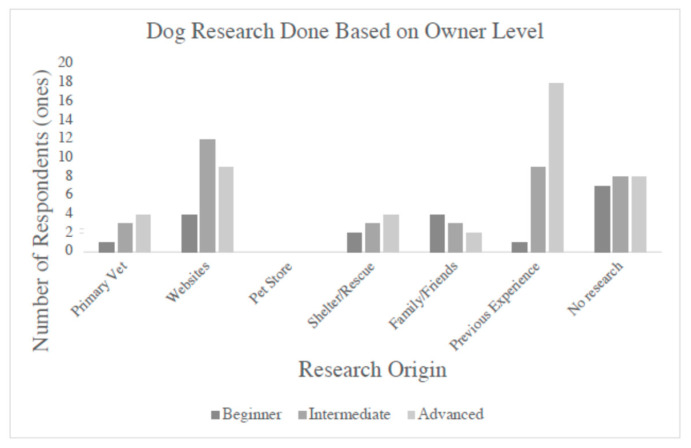
Research carried out prior to dog ownership. Compared to intermediate and beginner dog owners, advanced dog owners represented the highest number of respondents that had not conducted any type of prior research.

Multiple responses were not allowed for the questions regarding ownership level and research before ownership. The results from these questions are shown in Figure 1. Most of the participants, 44% (45/102), self-identified as being an advanced dog owner. The rest considered themselves to be intermediate, at 37% (38/102), and beginner first-time owners, at 19% (19/102). In response to the question regarding whether any research on dog ownership had been undertaken prior to ownership, 27% (28/102) of the dog owners believed they had enough experience and did not research prior to dog ownership, 23% (23/102) simply did not research owning a dog. Of those that undertook research, 25% (25/102) carried out research only on popular dog websites, 9% (9/102) used shelters/rescues as their source, 9% (9/102) obtained information from experienced family/friends, and 8% (8/102) obtained information from their primary veterinarian. Of the 45 advanced dog owners, 40% (18/45) believed they had enough experience, 18% (8/45) had not carried out any research, 20% (9/45) had gone to popular dog websites, 9% (4/45) had asked their primary vet, as well as 9% (4/45) asking at shelters/rescues, and finally, 4% (2/45) had researched by asking family and friends. This means that 57% (26/45) of the advanced owners had not carried out any type of research. For the intermediate-identifying dog owners, most had performed some type of research. Of the 38 owners, 32% (12/38) had used popular dog websites, and carrying out research by asking at local shelters/rescues and asking primary veterinarians and family and friends all represented 8% (3/38) of the intermediate dog owners. For those that had not researched, 24% (9/38) believed they had enough experience, and the remaining 21% (8/38) did not research at all. Finally, for the 19 beginner dog owners, most had carried out research, with 5% (1/19) researching through a primary veterinarian, 21% (4/19) through popular dog websites, 11% (2/19) through shelters/rescues, and 21% (4/19) through family/friends with experience. For those beginner owners that did not research, 5% (1/19) believed they had enough experience, and 37% (7/19) did not carry out any type of research. 

### 3.4. Primary Veterinarian Total 

Of these participants, the majority did have a primary veterinarian for their dog, specifically 90% (92/102). The remaining 10% (10/102) did not have a primary veterinarian. Of the 92 respondents that said yes to having a primary veterinarian, 18% (17/92) of them were beginner dog owners, 36% (33/92) were intermediate dog owners, and finally, 46% (42/92) were advanced dog owners. These percentages are reflective of the previously mentioned ratios of the ownership levels of the participants in general. 

### 3.5. The Primary Veterinarian and Outside Animal Contact Group 

Of the dogs that had a primary veterinarian, 58% (53/92) had contact with animals outside of the home. A total of 53 dogs had further social interactions, which included 64% (34/53) of them receiving professional nail trims, 68% (36/53) being professionally groomed, 19% (10/53) occasionally boarding, and 45% (24/53) having some type of access to the outdoors for their housing. At the beginning of the survey, 81% (43/53) responded that they did not live in an area with a canine leptospirosis outbreak (despite them confirming that they lived in Los Angeles County), and 19% (10/53) they did. 

Most of the owners, at 64% (34/53), were not familiar, however, with leptospirosis, and 36% (19/53) responded that they were familiar. Of those familiar with leptospirosis, 42% (8/19) learned about the disease from their primary veterinarian, 16% (3/19) learned about the disease from family/friends, 11% (2/19) from a news outlet, 5% (1/19) from an online forum, 5% (1/19) learned about leptospirosis from a shelter, and 21% (4/19) listed their source as other. The other sources included working in veterinary medicine and school. There was an increase in no response when they were asked whether they were familiar with the signs of leptospirosis, at 79% (42/53), while 21% (11/53) did know the signs. The percent for no response increased to 83% (44/53) when they were asked whether they knew that leptospirosis was a zoonotic disease, versus the 17% (9/53) who did. Finally, the highest percent at 89% (47/53) said they were unaware of how to properly alert to a potential case of leptospirosis versus the 11% (6/53) who were. 

Although most of the 53 respondents with a primary veterinarian were not familiar with the characteristics of leptospirosis, 62% (33/53) were aware of the vaccine for leptospirosis being available in their area, and 38% (20/53) were not. The vaccine rate for this group was only 25% (13/53). Those who chose to vaccinate mostly did so after consulting with their primary veterinarian, specifically 85% (11/13). The other two respondents who vaccinated their dog either did so on advice from family/friends, 8% (1/13), or based the decision on information from online news outlets, 8% (1/13). For the 75% (40/53) who had dogs that were not vaccinated against leptospirosis, 15% (6/40) opted for this choice after consulting with their primary veterinarian. The 80% (32/40) majority of those who did not vaccinate their dogs reported being unfamiliar with the disease until participating in the survey. The final 5% (2/40) that did not have their dog vaccinated against leptospirosis did not feel at the time of the survey their dog needed to be vaccinated but did have plans to vaccinate later on. 

### 3.6. The Primary Veterinarian and No Outside Contact Group

The dogs reported that did not have contact with outside animals accounted for 42% (39/92) of those with a primary veterinarian. Of these 39 dogs, 67% (26/39) have their nails trimmed by a professional, 69% (27/39) are professionally groomed, 21% (8/39) sometimes board, and 49% (19/39) are housed with some type of access to the outdoors. At the beginning of the survey, 82% (32/39) answered that they did not live in an area of a canine leptospirosis outbreak versus the 18% (7/39) who chose the response that they did live in an outbreak area. Out of these owners, 69% (27/39) were not familiar with leptospirosis and 31% (12/39) were. Of those familiar, 17% (2/12) had learned about the disease from their primary veterinarian, 8% (1/12) from family and friends, 33% (4/12) from an online news outlet, and 17% (2/12) had learned about leptospirosis from an online forum. A total of 25% (3/12) had a different source listed, like working in the vet medicine industry or school. 

When asked about the signs for leptospirosis, 74% (29/39) did not know the signs to look for and 26% (10/39) did, while 69% (27/39) did not know it was zoonotic and 31% (12/39) did. A total of 79% (31/39) of these respondents did not know how to alert to a possible case of leptospirosis. Meanwhile, 21% (8/39) said they did. A total of 54% (21/39) of the respondents did know whether there was a vaccine available in their area for leptospirosis versus the 46% (18/39) who did not. Of these dogs, 21% (8/39) were currently vaccinated, with 75% (6/8) of them being vaccinated due to recommendations from their veterinarian and 12% (1/8) due to family/friends and 12% (1/8) news/online sources. Of the 79% (31/39) who were not vaccinated, 13% (4/31) of the respondents indicated their decision was based on their primary veterinarian not recommending it, 3% (1/31) of the respondent did not feel like their dog needed the vaccine, 74% (23/31) of the respondents were not familiar with the disease until the time of the survey, and the remaining 10% (3/31) planned to vaccinate their dogs later. Note that 90% (83/92) were taken for yearly wellness exams and 96% (88/92) were up to date on their vaccines. 

### 3.7. No Primary Veterinarian Total

The 10% (10/102) dog owners that did not have a primary veterinarian were broken up into 20% (2/10) of them being beginner dog owners, 50% (5/10) being intermediate dog owners, and 30% (3/10) being advanced dog owners. 

### 3.8. The No Primary Veterinarian and Outside Animal Contact Group

The group of dogs with no primary veterinarian could be broken down further with 40% (4/10) having contact with outside animals and 60% (6/10) reporting no contact with any outside animals. Of the dogs with no outside animal contact, 75% (3/4) receive professional nail trims, 50% (2/4) have access to the outdoors, 50% (2/4) sometimes board, and 75% (3/4) are professionally groomed. When the owners were first asked about whether they resided in a canine leptospirosis outbreak area, 100% (4/4) responded with no. The respondents from this group answered no at a rate of 100% (4/4) to being familiar with leptospirosis, whether they were aware it is zoonotic, the signs to look out for, and whether they knew how to alert to a case. However, 25% (1/4) knew the leptospirosis vaccine was available versus 75% (3/4) that did not. One hundred percent (4/4) of these respondents did not have their dogs vaccinated due to them not being familiar with the disease until the time of the survey.

### 3.9. The No Primary Veterinarian and No Outside Animal Contact Group

Of the remaining 60% (6/10) of the dogs that do not have contact with outside animals, 50% (3/6) receive professional nail trims, 50% (3/6) have access to the outdoors, and 67% (4/6) are taken to be professionally groomed. At the beginning of the survey, 33% (2/6) responded that they did live in an area of an outbreak, while 67% (4/6) reported that they did not. Of the owners from this group, 33% (2/6) of them were familiar with leptospirosis in dogs, and the remaining 67% (4/6) were unfamiliar. The 33% (2/6) who were familiar with it cited their primary veterinarian, at 50% (1/2), and listed experience in the veterinary industry, at 50% (1/2), as their source for knowing about leptospirosis in dogs. The 33% (2/6) and 67% (4/6) ratio remained the same for the following survey questions regarding the signs to look out for and leptospirosis being zoonotic but dropped to 17% (1/6) for owners who knew how to report a case and 83% (5/6) who did not know how. A total of 50% (3/6) of the owners knew whether there was a vaccine available near them, and 50% (3/6) did not. Only 17% (1/6) had their dogs vaccinated due to reading online sources. Of the 83% (5/6) of owners who had not vaccinated their dogs, 20% (1/5) wanted to vaccinate them at a later time, and 80% (4/5) reported being unfamiliar with the disease until the time of the survey. Note that 20% (2/10) of the total dogs are taken for yearly wellness exams and 60% (6/10) are up to date on their vaccines. 

### 3.10. The Vaccination Rate Totals

The leptospirosis vaccination rates based on total respondents without considering the ownership level or primary veterinarian were as follows: 22% (22/102) dogs vaccinated and 78% (80/102) not vaccinated. The decision for owners to vaccinate was split as follows: 77% (17/22) made this choice after consulting with their primary veterinarian, 9% (2/22) due to discussing it with family/friends with experience, and 14% (3/22) after reading news/online sources about the disease. For those who chose not to vaccinate, their decisions were split as follows: 12% (10/80) made this decision after consulting with their primary veterinarian, 8% (6/80) planned to vaccinate their dogs later, 1% (1/80) believed their dog did not need to be vaccinated at all, and 79% (63/80) were not familiar with the disease until the time of the survey. The vaccination rates based on primary veterinarian alone were 23% (21/92) for those that had a primary veterinarian and 10% (1/10) for those that did not have a primary veterinarian. 

### 3.11. Vaccination Rate Based on Ownership Level

**Figure 2:**
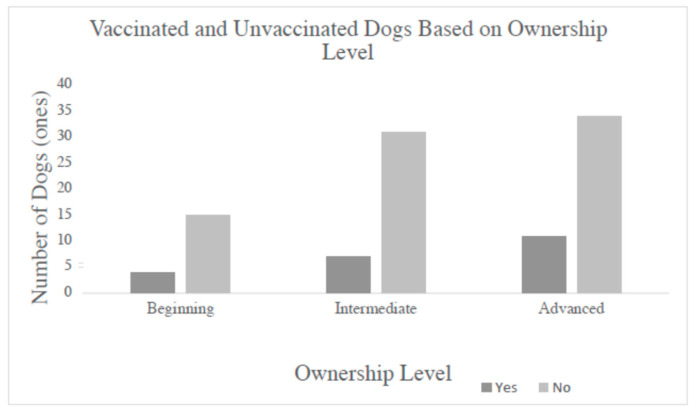
Unvaccinated for leptospirosis versus vaccinated for leptospirosis based on the level of ownership. Each group displayed similar responses in terms of the percentage vaccinated or unvaccinated regardless of ownership level. Data shown include all respondents (n = 102).

The leptospirosis vaccination rate totals based on dog ownership level were as follows: 21% (4/19) vaccinated versus 79% (15/19) that did not vaccinate for beginner dog owners, 18% (7/38) that vaccinated versus 82% (31/38) that did not vaccinate for intermediate dog owners, and 24% (11/45) that vaccinated versus 76% (34/45) that did not vaccinate for advanced dog owners. The dogs with a primary veterinarian and belonging to a beginner dog owner had a leptospirosis vaccination rate of 24% (4/17), while this was 21% (7/33) for dogs with intermediate dog owners and 24% (10/42) for dogs belonging to advanced dog owners. For the dogs without a primary veterinarian, their vaccination rates were 0% (0/2) for beginner dog owners, 40% (2/5) for intermediate dog owners, and 33% (1/3) for advanced dog owners (see Figure 2). 

### 3.12. Chi Square Analysis 

A chi square analysis was undertaken for the group “primary veterinarian and contact with outside animals”. This analysis was to determine whether leptospirosis vaccination status and the availability of the vaccine had an association. The *p*-value was 0.001 (*p* ≤ 0.05), which indicated statistical significance. This means that the null hypothesis of the two being independent could be rejected. A second chi square analysis was performed for the group “primary veterinarian with no contact with outside animals”, this analysis evaluated whether leptospirosis vaccine availability in the area was associated with the leptospirosis vaccination status. The *p*-value was 0.097 (*p* > 0.05), which indicated that there was no association, and the analysis failed to reject the null hypothesis, or the variables were independent of each other. The selection of the “primary veterinarian and contact with outside animals”, n = 53, and “primary veterinarian with no contact with outside animals”, n = 39, groups was based on the available sample sizing and the risk factors they presented. 

## 4. Discussion 

Owners with a primary veterinarian and whose dogs participated in social activities and were exposed to outside animals had a higher leptospirosis vaccination rate and knowledge of the disease compared to these levels in owners who did not have a primary veterinarian and participated in less or no social activities. It is also worth noting that the number of respondents (10) that reported that they did not have a primary veterinarian does not represent most of these dog owners. Thus, it is difficult to conclude the direct cause of opting out of the vaccine when there was still a poor rate among dogs with a primary vet. 

The results of our survey differed in comparison to those of other similar studies. A study conducted in 2022 to identify the vaccination rates for non-core vaccines found inconsistent results amongst clinics. The dogs in said study were up to date on all of their core vaccines, with leptospirosis vaccination ranked the highest in terms of median clinic vaccination at a rate of 70.5% out of the 2,798,875 dogs involved in this study. However, individually, the clinics had vaccination rates for leptospirosis that ranged from 0% up to 100% [10]. 

The dog owners that took part in this present survey were primarily women, with only 10% of the respondents identifying as men and 1% identifying as non-binary. Similarly, in a survey regarding canine blood donation, a majority of women were found to have participated in the survey [16]. In this survey, the targeted social media group into which the survey link was posted had more of an evenly distributed membership base of men and women, so one possible assumption is that the women felt more inclined to participate than the men belonging to this group. The exact demographics of the group were not analyzed, so there is the possibility that the group did have more women than men. The same logic could be applied to the age groups that participated in the survey since even though there were respondents from all groups present in the survey, the majority were between 25 and 34 years old. It could be that the group was composed mostly of dog owners in this age group. The focus on a single online group was intended to have better control over ensuring that individuals from the targeted community (those residing where there was a canine leptospirosis outbreak in the Los Angeles County area) participated. Additionally, the majority of these social groups had rules that restricted what could be posted. Many Facebook groups did not allow for specific marketing or self-promotion posts, and therefore the survey link would have violated these guidelines. Posting within a single group ensured that the post would not be taken down and the survey results would not be lost or compromised. 

When analyzing the results based on the level of dog ownership, most of the participants in the survey identified as advanced dog owners. The questions regarding the level of ownership aimed to determine what type of preparation had been taken before obtaining a dog. Most respondents claimed to have enough experience and did not perform any research. When broken down further, the advanced dog owners mainly fell into this group. When research on adopting a dog was performed, regardless of ownership level, popular dog websites were the favored source. There was a similar finding during a survey on raw meat based diets, where owners primarily used popular dog websites for nutritional information when creating a raw diet for their dog [17]. In both this survey on leptospirosis and the survey by Morelli, the exact dog websites used by the owners was not a question asked to the respondents, which means there may have been a great variety of information that was accessed, with or without credibility. From this type of question, the idea that advanced dog owners believe they have enough knowledge or experience and do not look to extend their knowledge further when adopting is an assumption that can be deduced from the collected responses. Advanced dog owners may be lacking knowledge in several areas but, with their belief in their previous experience, may fail to acquire deeper knowledge and understanding of dogs’ needs and health. 

When comparing the leptospirosis vaccination rates between all three levels of dog ownership, regardless of primary veterinarian, activities, or contact, the advanced dog owners only had a marginally higher leptospirosis vaccination rate, at 24%, versus the beginner dog owners, who had a vaccination rate of 21%. If having a primary veterinarian is included in this rate, then the rate between the beginner and advanced dog owners is equivalent, at 24%. Based on these results, it can be assumed that the self-proclaimed level of dog ownership does not affect the rate at which dog owners are vaccinating against leptospirosis. When analyzing the results based on level of ownership, the grouping was not further broken down into specific categories of social activities versus no social activities, as the number of respondents would not have been a large enough group when already divided into three sub-sections. Although it was not based on ownership level, another study conducted on the vaccination rates and causes of owner attitudes towards vaccinating their dogs also yielded low leptospirosis vaccination rates. The rate for leptospirosis vaccination was 50.1% (1941/3874), which was still low when compared to the other vaccines measured, such as rabies, at 86.3% (3343/3872), and distemper/parvo, at 75.5% (2.915/3863). This study also noted that recommendation by their primary veterinarian was the primary influence on these rates [18]. When a chi square analysis was performed to determine whether there was an association between having the vaccine available in the area and vaccination rate, there were mixed results. For the group that had a primary veterinarian and dogs that had contact with outside animals, the *p*-value was 0.001. This indicates that having the vaccine available increases the rate of vaccination (in dogs in contact with outside animals). However, when the same chi square analysis was carried out for the group that had a primary veterinarian and dogs with no contact with outside animals, the *p*-value was 0.097. It is possible that since the group with outside contact includes dogs potentially at a higher risk of leptospirosis (due to their contact with other animals), having a local leptospirosis vaccine influences owner decisions to vaccinate them [19]. In the other group, these dogs do not have contact with outside animals, and therefore they may be at lower risk of exposure to leptospirosis. Since there is an assumed decreased risk, then the leptospirosis vaccine’s availability would not matter to this group of owners. 

A study looking at dog vaccination rates and owner attitudes to attending vaccine clinics found that 70% of the respondents to the questionnaire felt that clinic accessibility needed to be addressed, among other issues [20]. Although this study took place in Grenada, it does demonstrate similar findings in that the owners were less likely to have their dogs vaccinated if there were issues with vaccine accessibility [20]. The chi square analysis carried out for this survey indicated that for at least one group, the availability of the leptospirosis vaccine had a significant association with leptospirosis vaccination status. These results could help to identify possible factors that affect the rates of leptospirosis vaccination among dog owners. If the availability of the vaccine is one of these, then increasing the number of clinics that carry the leptospirosis vaccine in the area could potentially increase leptospirosis vaccination rates. This is due to the analysis for at least the higher-risk group of dogs with outside contact showing its dependent relationship with their leptospirosis vaccine status and whether the vaccine was readily available. For the group that showed an independent relationship, these results could help to eliminate factors unrelated to leptospirosis vaccination status. 

There was willingness among almost all of the respondents to further their education about their dogs’ wellbeing and health, as shown by a follow-up question about their desire to carry out independent research. Out of the total respondents, 76% (78/102) said they would like to do more research on leptospirosis. By providing the correct information in the sources that are most commonly used by dog owners, there is a greater chance of spreading awareness to the community. As demonstrated by the results of this survey, the areas that would be most impactful for reaching dog owners would be popular dog websites, primary veterinarians, and breeders. Another way to increase awareness regarding leptospirosis would be to have local officials spread information, as most of the respondents did not believe their local sources were doing enough to spread awareness about current outbreaks. This work could be carried out by local county officials or city officials or in combination with local animal shelters. 

One occurrence of how investigators looked to determine a possible human outbreak of leptospirosis during an outbreak in dogs in Arizona was reported in a research article from 2019 [13]. Cases of leptospirosis in dogs were identified and reported to the state veterinarian to be identified further as confirmed, probable, or suspected [13]. As the dog cases were under investigation, human serological tests were performed concurrently in those who were determined to have had exposure to any of the confirmed, probable, or suspected cases. This was carried out by contacting dog owners, veterinary facilities, and boarding facilities to inform them of the outbreak. Reports were also received regarding symptomatic providers or physicians treating symptomatic patients [13]. This investigation was the first of its kind in the United States in which testing was carried out simultaneously while the outbreak in dogs occurred [13]. This study found that all individuals tested seronegative, with one-quarter of them having come into contact with urine or blood from infected dogs at some point during the outbreak. However, even with these negative results, the investigators did note that it was most likely the *Leptospira* serovar Canicola that was infecting the canine population, which is a canine-adapted strain [13].

If future studies were to be conducted, a strain such as Icterohaemorrhagiae could produce more positive results, as it is thought to be more transmissible to humans. The research highlighted how safety guidelines to prevent outbreaks were not consistent with the actual day-to-day work that is undertaken at the facilities participating in said study [13]. Specimens were to be treated as infectious until evidence and tests confirmed otherwise; however, the reporting of glove and other protective equipment use was seen to be more consistent for blood handling than when urine was involved [13]. This research also emphasized how animal care providers, especially veterinary technicians, should receive the most information, as they are at the highest risk of being exposed to potentially infected bodily fluids [13]. The public health response also plays a role when considering a leptospirosis outbreak in a dog population [13,16]. People who had come in contact with the disease should be notified, provided with education about the disease, informed of the symptoms, and encouraged to seek medical treatment if necessary. 

### Limitations

The first limitation encountered was with the gender of the respondents. Out of 102 respondents, 92 (90%) of them identified as a woman. Thus, the results could be interpreted as a reflection of women dog owners and not owners in general. Similarly, the majority of the participants were in the 25–34 year-old range; this does not reflect the overall population of Los Angeles County and may over represent the opinions in this age group. 

Sample size was also an issue that occurred in the survey. The total number of respondents was 102. Based on other studies conducted, this number was on the smaller side. The sample of 134 taken in a study on the risk and predictive factors for leptospirosis in dogs was the minimum size noted for similar research [21]. Another survey looking at owners’ motivations and fears in blood donation had a sample size of 207 [16]. However, some online surveys, such as one conducted on the influence of veterinarians on vaccination rates, had sample sizes as large as 1,480 [11]. Another large survey sample size was applied in a study on dog owners’ purchasing habits for grain-free food, which produced results from 3,298 respondents [22].

Interpretation of the survey questions was also a limiting factor. Questions such as the question on the source of research about dog adoption were limited to one choice. Multiple sources may have been used by the owners. In addition, some of the questions may have been too vague. Many chose “popular websites” as a source of information; however, there were no specific sites given. Therefore, respondents’ perspectives could have altered the results. 

The survey length could also have been a potential limiting factor. The survey consisted of five question sets with a total of 47 questions, including the question obtaining the consent of the respondents. Although the survey took an estimated 5 min, based on the SurveyMonkey software’s analysis, this still may have been too long for some respondents. Given that there were over 200 surveys that had to be discarded due to incompletion, there is the possibility some stopped because of its length and not due to them retracting their consent. 

To improve this survey, a follow-up questionnaire with a greater pool of respondents to create larger groupings would yield more accurate results. In addition, the questions could be clarified further. The wording could be altered and definitions provided. It is also possible that the owners obtained information from multiple sources, and we asked the owners to pick the best answer. Future research can be conducted to determine the most popular dog websites to include, and references to other studies can be used to determine dog ownership levels or experience. Another improvement to the survey would be ensuring a balance between the genders of the respondents so that results were more representative of all dog owners. A future survey could also be more precise; this means it would include only the necessary questions that would be used for analyzation. Additionally, a shorter survey could possibly decrease the number of incomplete surveys received and condense the amount of data.

Canine leptospirosis is a zoonotic disease that has the potential to cause outbreaks in humans, wildlife, and domesticated dogs [23]. This bacterial disease can be found in the environment, particularly in standing water, such as puddles or swimming areas, and in the urine of infected animals [23]. Consequently, there are multiple ways in which humans can contract it. The lifestyles of both owners and canines can play a role in the risk level of a possible infection. Spending time outdoors near potentially infected water sources; bringing dogs to social areas such as groomers, boarding facilities, and even animal hospitals; and finally, being unaware of the signs of a leptospirosis infection can all increase the risk of contracting leptospirosis [23]. Although transmission from dogs to humans is considered relatively low, without having at least basic knowledge of the signs of a possible leptospirosis infection, the risk of contamination and human infection could possibly increase. Dog owners who are not educated on canine leptospirosis may also not be aware of the available vaccines. Therefore, dogs participating in an at-risk lifestyle could be left exposed to contracting leptospirosis. The goal of this research was to conduct a survey to determine whether having a primary veterinarian and social lifestyle led to a greater response from dog owners to canine leptospirosis given the recent outbreak of the disease in the southern Californian communities within Los Angeles County. 

## 5. Conclusions

From the data collected, it could not be determined whether having a primary veterinarian and social life led to a higher response rate in terms of vaccination status or knowledge of canine leptospirosis. This was due to the sample size of the respondents who did not have a primary veterinarian being considerably smaller than that of those who did have a primary veterinarian. In addition, these data are not a reflection of all dog owners in the Los Angeles County area since more women than men voluntarily participated in the survey, causing an unequal distribution. In any case, the overall results from all respondents showed that the majority were not aware of leptospirosis before participating in the survey. Furthermore, an even greater number of respondents did not know what the signs were or how a case would be reported. Most respondents agreed that they required more in terms of warnings from local officials when zoonotic outbreaks occurred in their area. 

Since the conclusion of the outbreak, Los Angeles County has reported a total of 13 dogs that have died from leptospirosis. Data on the treatment of the infected dogs were available for 164 cases and showed that 55% (91/164) of these dogs were hospitalized. After this outbreak occurred, the county suggested the leptospirosis vaccine be considered a core vaccine for any dog in the Los Angeles County area [12]. Although dogs were the main form of transmission, officials from the county advised that wildlife still posed a threat in terms of the other various serovars. Veterinarians were also advised to speak more to clients to educate them about their dog’s risk of contracting leptospirosis and about vaccinating [12].

## Figures and Tables

**Table 1:** Gender and age group of the respondents (% rounded to nearest whole number).

Gender	18–24 YR	25–34 YR	35–44 YR	45–54 YR	55–64 YR	65+ YR	Total % n = 102
Men	2 (2%)	7 (7%)	0 (0%)	0 (0%)	0 (0%)	1 (1%)	10%
Women	11 (11%)	36 (35%)	12 (12%)	16 (15%)	12 (12%)	4 (4%)	89%
Non-binary	0 (0%)	1 (1%)	0 (0%)	0 (0%)	0 (0%)	0 (0%)	1%
Total %	13%	43%	12%	15%	12%	5%	100%

**Table 2:** Sex and age group of the dogs (% rounded to nearest whole number).

Sex	0–2 YR	3–4 YR	5–6 YR	7–8 YR	9–10 YR	11+ YR	Total % n = 102
Altered Male	22 (21%)	4 (4%)	8 (8%)	6 (6%)	5 (5%)	8 (8%)	52%
Unaltered Male	4 (4%)	1 (1%)	2 (2%)	1 (1%)	1 (1%)	0 (0%)	9%
Altered Female	9 (9%)	6 (6%)	2 (2%)	3 (3%)	7 (7%)	9 (9%)	35%
Unaltered Female	3 (3%)	0 (0%)	1 (1%)	0 (0%)	0 (0%)	0 (0%)	4%
Total %	37%	11%	13%	10%	13%	17%	100%

## Data Availability

Aggregated data that maintains the anonymity of the respondents are included in the Supplementary Materials.

## Supplementary Materials

The following supporting information can be downloaded at: https://www.mdpi.com/article/10.3390/vetsci12020119/s1, File S1: Dog Owner Demographics And Response To Recent Leptospirosis Outbreak Survey.
