# Supplementary material for: Evaluating a Response to a Canine Leptospirosis Outbreak in Dogs Using an Owner Survey
Source: Vet Sci. 2025 Feb 2;12(2):119. doi: 10.3390/vetsci12020119 (PMC11860354; doi:10.3390/vetsci12020119)
Supplement: Supplementary file 1 [file vetsci-12-00119-s001.zip › vetsci-3374040-supplementary.pdf]

SUPPLEMENTARY MATERIALS:
DOG OWNER DEMOGRAPHICS AND RESPONSE TO RECENT LEPTOSPIROSIS OUTBREAK SURVEY

Please read the consent information before beginning.

* 1. The Cal Poly Pomona Institutional Review Board has reviewed and approved for conduct this research involving human subjects under protocol IRB 21 – 200. By clicking the I agree option below, you are agreeing to electronically consent to taking this survey and providing the asked information. The purpose of this information is to be used in research to further understand the response to leptospirosis in dogs due to the current outbreak. These results will be aggregated and may be published. Confidentiality will be maintained with exceptions to the questions asked in the survey such as gender and age range. You may end your consent and exit the survey at any time. By selecting I disagree you do not consent to providing the asked information and can freely exit the survey. For any questions regarding this research or your consent rights please contact: syvillanueva@cpp.edu.

- I agree 100% (102/102)
- I disagree

Question Set 1: Primary Owner Background

Please answer the following background information to the best of your ability.

* 2. Gender: How do you identify?

- Man 10% (10/102)
- Woman 89% (91/102)
- Non-binary 1% (1/102)
- Prefer not to say
- Prefer to self-describe, below

Self-describe:

* 3. Select the age group of the primary owner that best fits. (age in years)

- 18-24 13% (13/102)
- 25-34 43% (44/102)
- 35-44 12% (12/102)
- 45-54 16% (16/102)
- 55-64 12% (12/102)
- 65+ 5% (5/102)

* 4. Do you have any children present in the home of your dog?

- Yes 35% (36/102)
- No 65% (66/102)

5. If yes to the previous question, please identify the age range of the youngest child (age in years).

- 0-5 33% (12/36)
- 6-10 22% (8/36)
- 11-15 19% (7/36)
- 16+ 25% (9/36)

* 6. Were you a resident in an area during a leptospirosis outbreak?

- Yes 19% (19/102)
- No 81% (83/102)

* 7. Are you the primary caregiver?

- Yes 85% (87/102)
- No 15% (15/102)

* 8. Are there multiple dogs present in the home?

- Yes 38% (39/102)
- No 62% (63/102)

* 9. What level of ownership would you consider yourself?

- Beginning first-time dog owner 19% (19/102)
- Intermediate owner (one to multiple dogs over a few years) 37% (38/102)
- Advanced owner (multiple dogs with numerous years of experience) 44% (45/102)

* 10. Was any research done prior to adopting your dog?

- o Yes, I consulted with my primary veterinarian 8% (8/102)
- o Yes, I researched on popular dog websites 25% (25/102)
- o Yes, I asked questions from my local pet store 0% (0/102)
- o Yes, I asked questions from my local shelter/rescue 9% (9/102)
- o Yes, I asked family/friends with experience 9% (9/102)
- o No, I believed I had enough experience 27% (28/102)
- o No, I did not do any research23% (23/102)

* 11. Do you have any other pets inside your home besides dogs?

- o Yes 34% (35/102)
- o No 66% (67/102)

Question Set 2: Dog Background

Please answer the following background information to the best of your ability based on ONE dog in your home.

* 12. What is the sex of the dog?

- o Male Altered 52% (53/102)
- o Female Altered 35% (36/102)
- o Male Unaltered 9% (9/102)
- o Female Unaltered 4% (4/102)

* 13. Please select the age range that best fits the dog. (age in years)

  - 0-2 37% (38/102)
  - 3-4 11% (11/102)
  - 5-6 13% (13/102)
  - 7-8 10% (10/102)
  - 9-10 13% (13/102)
  - 11+ 17% (17/102)

* 14. Where was your dog obtained from?

  - Rescue/Shelter 38% (39/102)
  - Animal hospital 3% (3/102)
  - Family/Friend 16% (16/102)
  - Breeder 38% (39/102)
  - Found lost dog 5% (5/102)

* 15. Does your dog have a primary veterinarian?

  - Yes 90% (92/102)
  - No 10% (10/102)

* 16. Is your dog housed with a companion?

  - Yes, one 22% (22/102)
  - Yes, multiple 21% (21/102)
  - No 58% (59/102)

* 17. Does your dog often show positive social behaviors? (i.e. playing, body posture, affection, etc.)

- o Yes 98% (100/102)
- o No 2% (2/102)

* 18. Does your dog often show negative social behaviors? (i.e. biting, humping, people/dog reactive, etc.)

- o Yes 33% (34/102)
- o No 67% (68/102)

* 19. Does your dog show frequent signs of illness? (i.e. lack of appetite, lethargy, vomiting, etc.)

- o Yes 4% (4/102)
- o No 96% (98/102)

Question Set 3: Husbandry

Please answer the following questions regarding care to the best of your ability.

* 20. How is your dog housed?

- o Always outside, shelter provided 2% (2/102)
- o Always outside, no shelter provided 0% (0/102)
- o Always inside 53% (54/102)
- o Both inside and outside, shelter provided 43% (44/102)
- o Both inside and outside, no shelter provided 2% (2/102)

* 21. How often is your dog fed?

  - o Once daily 12% (12/102)
  - o Twice daily 79% (81/102)
  - o More than twice a day 9% (9/102)

* 22. Does your dog have constant access to water?

  - o Yes 100% (102/102)
  - o No 0% (0/102)

* 23. Is your dog provided a store brand or prescription diet?

  - o Store brand 83% (85/102)
  - o Prescription 17% (17/102)

* 24. Do you provide your dog with any supplements?

  - o Yes 40% (41/102)
  - o No 60% (61/102)

* 25. Do you take your dog to be groomed?

  - o Yes 69% (70/102)
  - o No 31% (32/102)

* 26. Is your dog provided with enrichment? (i.e. chewing toys, bedding, dog parks, etc.)

  - o Yes 100% (102/102)
  - o No 0% (0/102)

* 27. Do you board your dog?

  - o Yes, regularly 0% (0/102)
  - o Yes, as needed 20% (20/102)
  - o No 80% (82/102)

Question Set 4: Health

Please answer the questions regarding your dog's health to the best of your ability.

* 28. Do you take your dog for annual wellness exams?

  - Yes 83% (85/102)
  - No 17% (17/102)

* 29. Is your dog currently up to date on vaccinations?

  - Yes 92% (94/102)
  - No 8% (8/102)

* 30. Do you take your dog for nail trims?

  - Yes, regularly 27% (28/102)
  - Yes, as needed 37% (38/102)
  - No, I cut them myself 24% (24/102)
  - No, I never cut my dog's nails 12% (12/102)

* 31. Does your dog suffer from any chronic conditions requiring regular veterinary visits?

  - Yes 9% (9/102)
  - No 91% (93/102)

* 32. Do you have any health insurance for your dog?

  - Yes 17% (17/102)
  - No 83% (85/102)

* 33. Has your dog had any emergency veterinary visits in 2021?

  - Yes 18% (18/102)
  - No 82% (84/102)

* 34. Does your dog have any contact with animals outside your home?

- Yes 56% (57/102)
- No 44% (45/102)

Question Set 5: Leptospirosis

Please answer the questions regarding leptospirosis to the best of your ability.

* 35. Are you familiar with leptospirosis in dogs?

- Yes 32% (33/102)
- No 68% (69/102)

36. If yes to the previous question, where did you learn about leptospirosis from?

- My primary veterinarian 33% (11/33)
- Family/Friend 12% (4/33)
- Online news outlet 18% (6/33)
- Online forum 9% (3/33)
- Shelter/Rescue 3% (1/33)
- Other (please specify)

* 37. Do you know the signs to look for with leptospirosis?

- Yes 23% (23/102)
- No 77% (79/102)

* 38. Were you aware that leptospirosis is zoonotic meaning it can be transferred to humans?

- Yes 23% (23/102)
- No 77% (79/102)

* 39. Do you know how to properly alert to a possible case of leptospirosis?

  o Yes 15% (15/102)

  o No 85% (87/102)

* 40. Is the leptospirosis vaccine available in your area?

  o Yes 57% (58/102)

  o No 43% (44/102

* 41. Is your dog currently vaccinated against leptospirosis?

  o Yes (go to #42 next) 22% (22/102)

  o No (go to #43 next) 78% (80/102)

42. What influenced your decision to vaccinate for leptospirosis?

  o I chose to vaccinate after consulting with my primary veterinarian 77% (17/22)

  o I chose to vaccinate after consulting with family/friends with dog experience 9% (2/22)

  o I chose to vaccinate after consulting with my local shelter/rescue

  o I chose to vaccinate after reading on the disease from news/online sources 14% (3/22)

43. What influenced your decision to not vaccinate for leptospirosis?

- I do not feel that my dog needs to be vaccinated after consulting with my primary veterinarian 12% (10/80)
- I do not feel that my dog needs to be vaccinated after consulting with experienced family/friends
- I do not feel that my dog needs to be vaccinated after consulting with my local shelter/rescue
- I do not feel that my dog needs to be vaccinated after reading news/online sources
- I do not feel that my dog needs to be vaccinated at this time, but plan to vaccinate later 8% (6/80)
- I do not feel that my dog needs to be vaccinated at all 1% (1/80)
- I was not familiar with leptospirosis in dogs until now 79% (63/80)

* 44. Leptospirosis can cause symptoms in dogs including fever, vomiting, diarrhea, joint pain, breathing difficulty, and kidney/liver failure amongst others. It is transmitted by contact with contaminated water/soil sources, occasionally bite wounds, or directly from infected urine from wild animals, pigs, horses, dogs, and cattle. Given this information, do you believe it is important to increase the availability of information regarding leptospirosis to dog owners and the community?

- Yes 95% (97/102)
- No 5% (5/102)

* 45. Do you feel your local authorities provide enough notice and information for leptospirosis during outbreaks?

- Yes 13% (13/102)
- No 87% (89/102)

* 46. The leptospirosis vaccine is not a core vaccine like a rabies, parvovirus, and distemper virus vaccine. Instead, it is usually recommended based on lifestyle. Do you think if more information was provided on elective vaccines, more owners would choose to vaccinate their dogs?

- Yes 92% (94/102)
- No 8% (8/102)

* 47. Do you feel this survey has made you want to become more informed about leptospirosis?

- Yes, I would like to do more research 76% (78/102)
- No, I already knew about it prior to this survey 11% (11/102)
- No, I am not interested 13% (13/102)
